# Supplementary material for: A novel model to quantify blood transit time in cerebral arteries using ASL-based 4D magnetic resonance angiography with example clinical application in moyamoya disease
Source: J Cereb Blood Flow Metab. 2025 Feb 13;45(6):1069–81. doi: 10.1177/0271678X251321640 (PMC11826826; doi:10.1177/0271678X251321640)
Supplement: sj-pdf-1-jcb-10.1177_0271678X251321640 - Supplemental material for A novel model to quantify blood transit time in cerebral arteries using ASL-based 4D magnetic resonance angiography with example clinical application in moyamoya disease [file sj-pdf-1-jcb-10.1177_0271678X251321640.pdf]

Supplementary Figures

Table S-1: Patient Data

Subject	Age	Sex	Diagnosis	Affected hemisphere angiographically	Symptomatic hemisphere	Previous surgery	Indication for MRI
1	19	Female	Moyamoya disease	Both	None	Indirect left	Possible new TIA/infarct
2	55	Female	Moyamoya disease	Both	Both	None	Pre-operative assessment
3	21	Female	Moyamoya syndrome (Down syndrome)	Left	None	None	Pre-operative assessment
4	60	Male	Carotid occlusion	Right	Right	Direct right	New TIA/infarct
5	34	Female	Moyamoya disease	Both	Right	Direct right	New TIA/infarct
6	44	Female	Moyamoya disease	Both	Left	Direct left	Post-operative assessment
7	18	Male	Moyamoya syndrome (Neurofibromatosis type I)	Both	Left	Indirect 1ef tand right	New TIA/infarct
8	14	Female	Moyamoya disease	Both	None	Direct left, indirect left/right	New TIA/infarct
9	27	Female	Moyamoya disease	Both	Left	None	Pre-operative assessment
10	69	Male	Moyamoya disease	Both	Left	None	New TIA/infarct
11	24	Female	Moyamoya disease	Both	Left	None	Pre-operative assessment
12	27	Male	Moyamoya disease	Both	Left	Direct and indirect left/right	Possible new TIA/infarct
13	42	Male	Healthy subject	None	None	None	Not applicable
14	30	Female	Healthy subject	None	None	None	Not applicable
Subjects were excluded due to artifacts in the 4D-MRA							

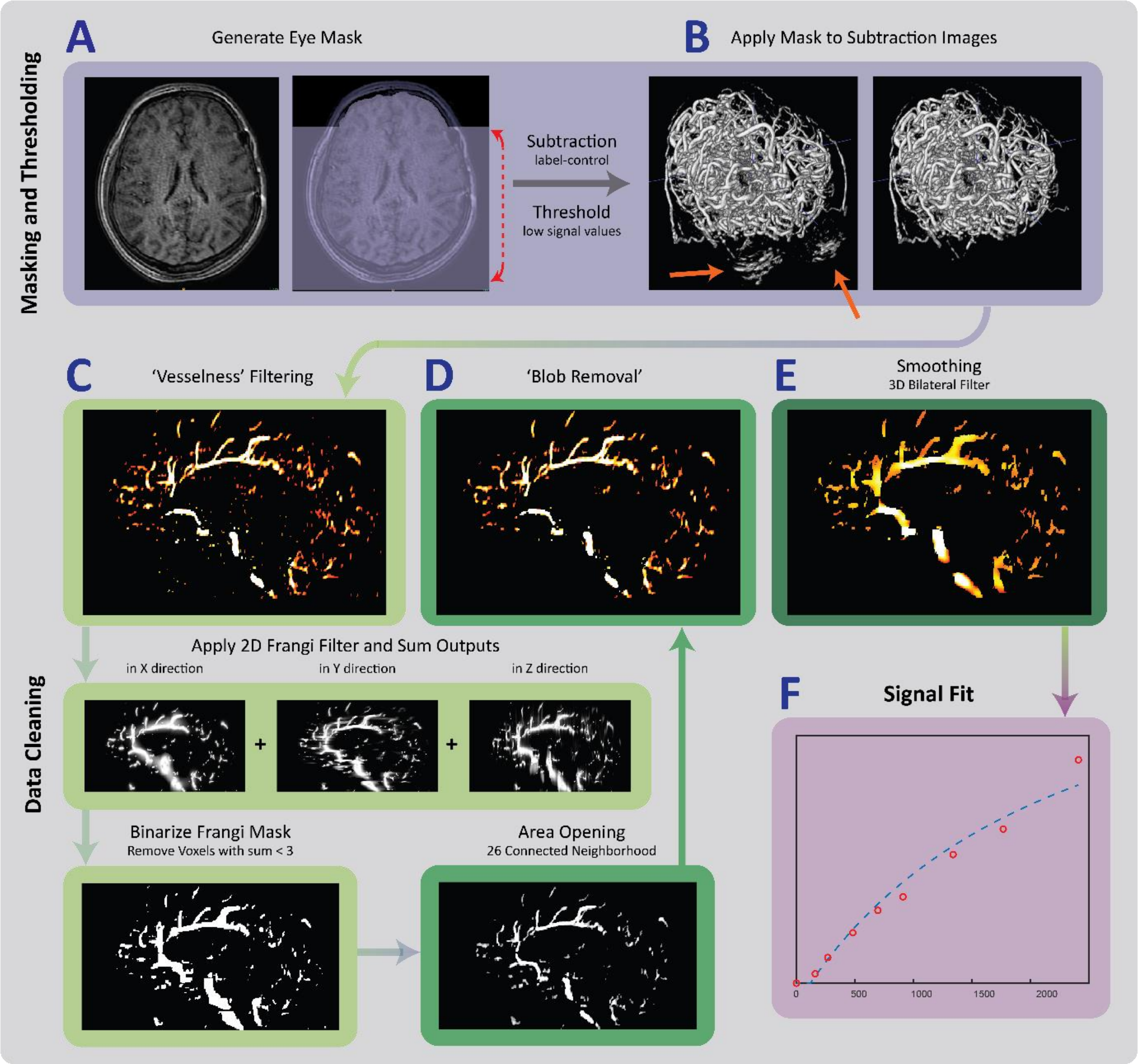

Figure S-1A: Magnitude images are initially brain-extracted using the FSL Brain Extraction Tool (BET) to create a whole-brain mask. Next, the posterior portion of this mask is modified to contain only 'ones' (highlighted in red). As a result, when applying this modified mask to the label-control magnitude images, only anterior extra-cranial voxels are removed, thereby preserving extracranial vessels and eliminating artifacts that originate in the ocular region (S-1B). Occasionally, subtle patient motion produces additional artifacts around the skull (e.g., skin movement). Although these artifacts do not corrupt vascular signals, they may be undesired and can be removed by reverting to the original whole-brain mask—with the trade-off of losing extra-cranial vessel information. S-1C: Once the inflow subtraction images are generated, an iterative 2D Frangi filter is applied in all three Cartesian directions. This filter enhances "vessel-like" structures in each direction and provides a corresponding binary mask. The three masks (XY, YZ, ZX) are summed, and only voxels with a value of '3' in the combined mask are retained. This resulting mask is then binarized and applied to the initial subtraction image to clean up vascular structures. Based on our observations, this approach captured more vascular structures than a 3D filter, though no systematic evaluation was performed. S-1D: Brain pulsation or disconnected vessel segments can lead to subtle artifacts in the subtraction images that may persist post-filtering. These "blob-like" artifacts are removed using an area-opening procedure in which any region with fewer than 26 connected voxels is excluded.S-1E: Finally, an edge-preserving smoothing step is performed via a bilateral filter that uses the vessel mask as a guide image, reducing

the risk of “broadening” the vascular tree. Nevertheless, some expansion of vessel boundaries is unavoidable. Note that alternatives exist for each of these preprocessing steps, offering opportunities for further refinement and optimization. S-1F: After completing the preprocessing, the piecewise saturation model (Equation 1; code available in the supplement) is fit on a voxel-wise basis.

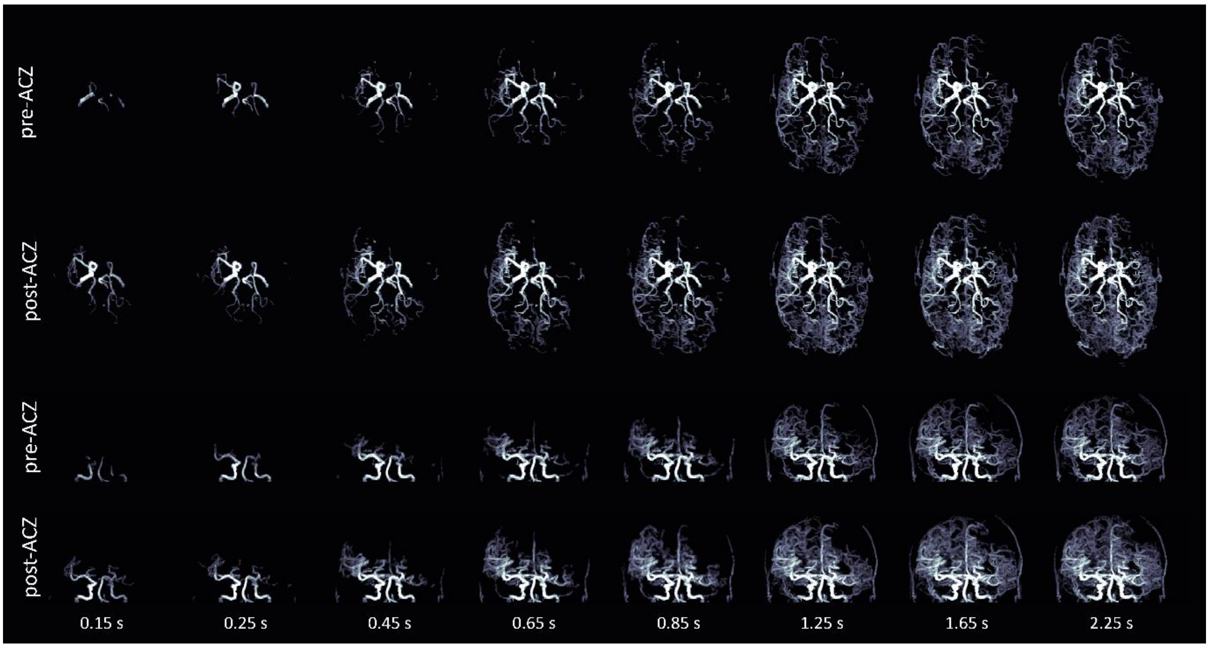

Figure S-2: The effect of Acetazolamide on the inflow signal. 4D-MRA images at progressive time-points both pre- and five minutes post-acetazolamide (ACZ) injection. Maximum intensity projections in the transverse (top) and coronal (bottom) orientation are shown. A supplementary PowerPoint file is included showing inflow videos derived from the piecewise model fit pre- and post-ACZ.

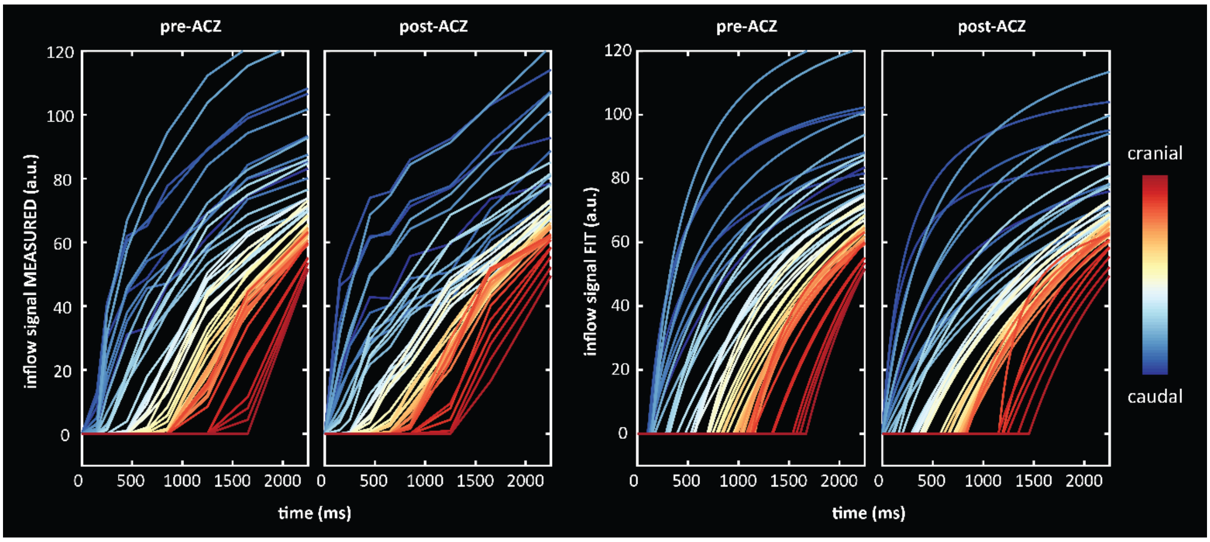

Figure S-3: measured (left) and fit (right) inflow curves (averaged per 3 slices) before and after ACZ injection in patient shown in figure 4 of manuscript and supplementary figure 1. Note the shortened arrival time.

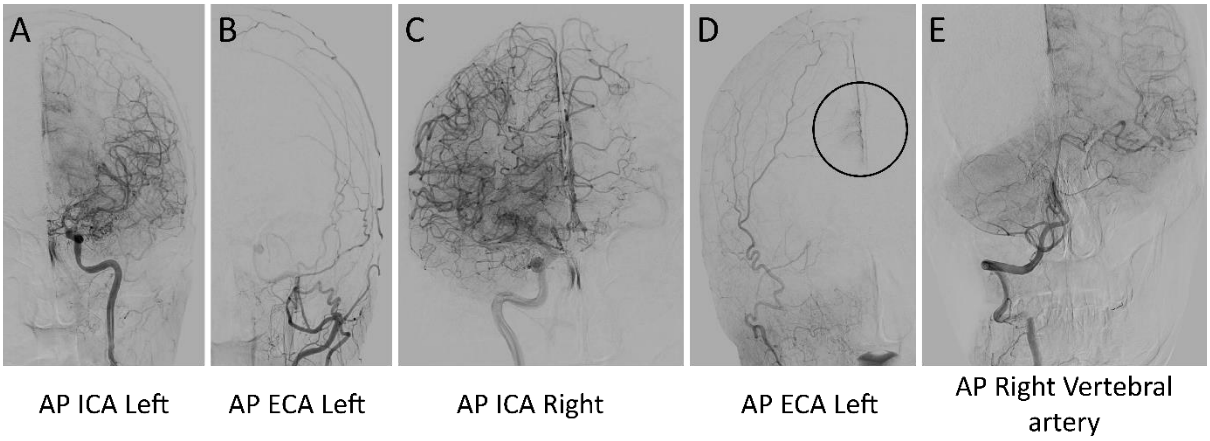

Figure S-4: Selected DSA images acquired at a referring hospital of the patient shown in figure 4 of the manuscript. A: AP image of the ICA on the left, showing occlusion of the distal ICA, the MCA and ACA cannot be identified, with extensive moyamoya collaterals; B: shows no naturally occuring ec-ic contribution from the left ECA; C shows the ICA on the right, with also extensive collateral formation around the distal ICA and no ACA identifiable. Contralateral flow of some bloodvessels; D: small contribution of the anterior territory form naturally occurring collaterals arriving from the ECA on the left side; E: contralateral filling of parts of the media territory on the left from the right vertebral artery.

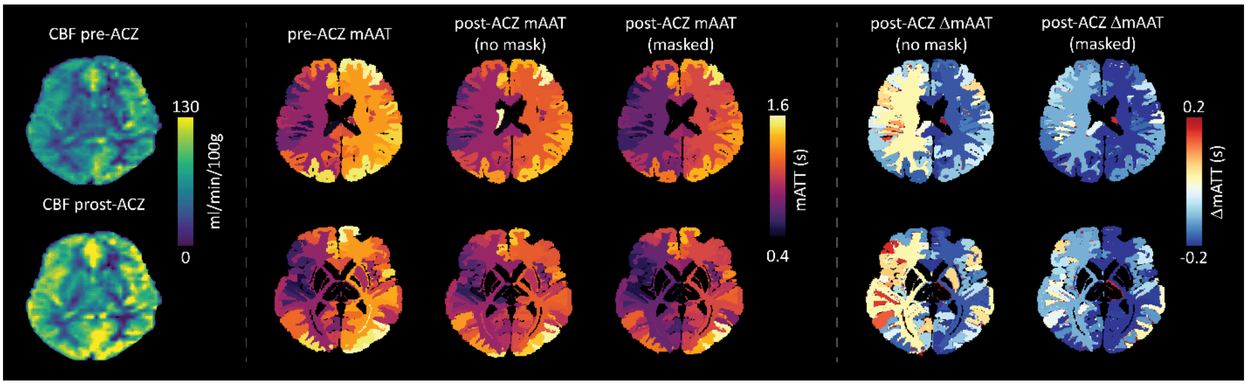

Figure S-5 – LEFT: Pre- and post-ACZ CBF maps in a patient shown in figure 5 of the manuscript. To investigate a possible source of the paradoxical mAAT values seen in the native atlas (contralateral to left-sided bypass), an additional analysis was performed in which the pre-ACZ vessel mask was used to mask the post-ACZ arrival time mask. After calculating the mean ROI values, the paradoxical arrival times on the unaffected side remained, however they became shorter than without the correction. This would suggest that the enhancement of distal vessels (and their associated longer mAAT) by the ACZ challenge can lead to increased arrival times in large regions where vessels with both short and long mAAT coincide. The observation that ΔATT in the contralateral hemisphere shows less decrease after ACZ injection may indicate a stronger contribution from leptomeningeal collaterals.

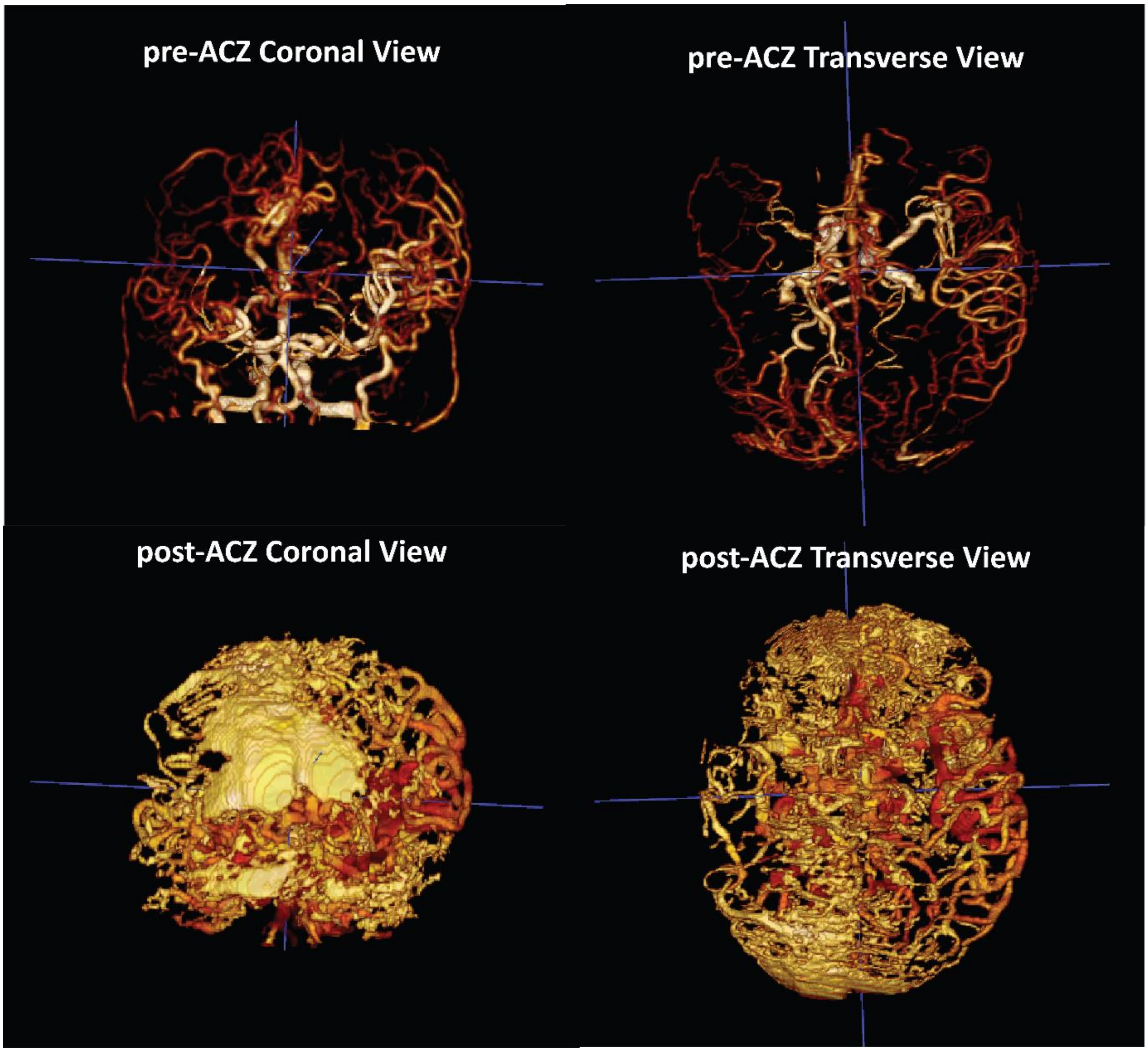

Figure S-5 – Subject 8, 14 year old female: Severe motion artifacts can lead to significant artifacts in ΔM images, which will inhibit quantification of mATT; TOP: the 8th time-point of the pre-ACZ ΔM image shows a successful acquisition with no visible artifacts. BOTTOM: severe motion between label and control images lead to widespread subtraction artifacts that could not be removed using filtering or other post-processing methods. This is not uncommon in young Moyamoya patients where holding still during long acquisitions can be challenging. In some cases, such artifacts are limited to edge regions of the brain (i.e. skin/skull), which we suggest may be mitigated by performing a brain extraction of the source images before subtraction and quantification – at the expense of losing information on extra-cranial vessels.

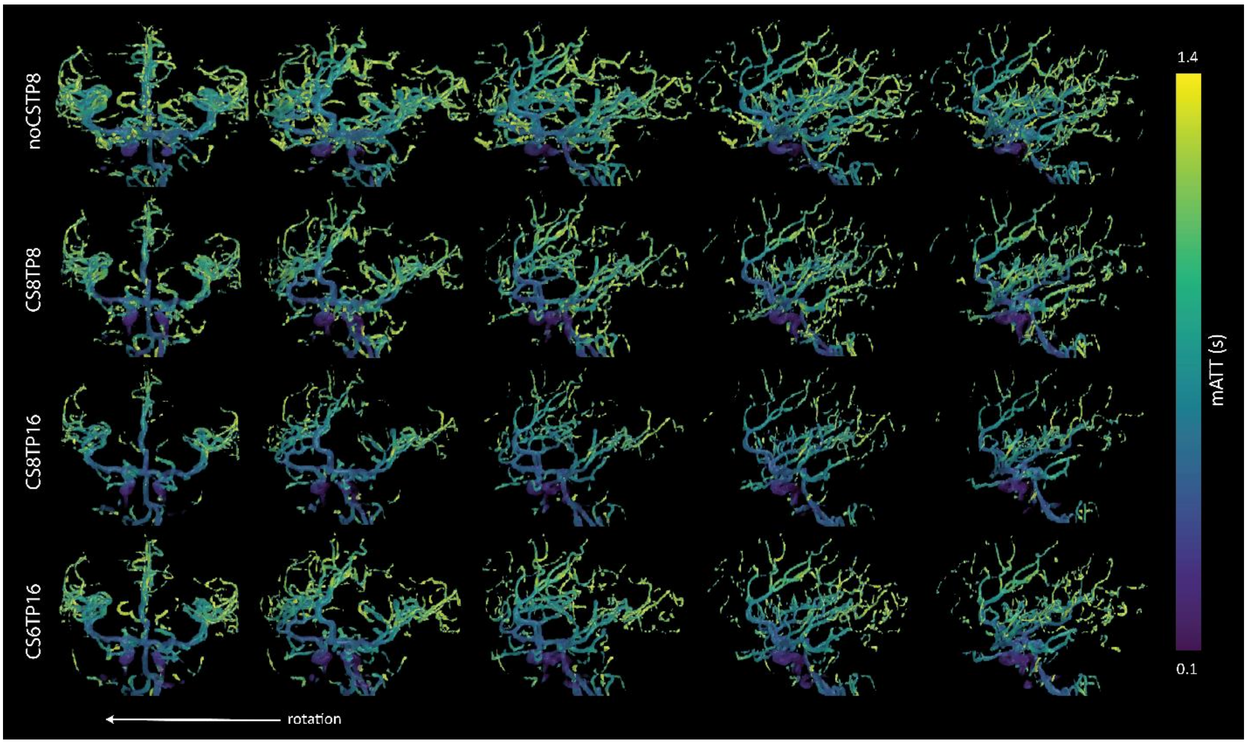

Figure S-6: The effect of increasing the number of time-points and accelerating the data acquisition using two different compressed sense (CS) factors. Increasing CS, can reduce scan time, but it comes at the cost of SNR at the distal vessels. A prospective study in healthy control subjects is warranted to determine the optimal balance of acceleration (to acquire more time points) and improved accuracy of the mAAT fit. The 'noCSTP8' scan (first row) was a reference scan with the same parameters as described in the main manuscript: 3D GRE readout, 8 time-points at 100, 200, 400, 600, 800, 1200, 1600 and 2200 ms, 75% keyhole central size, turbo factor=60, SENSE factor=3x1.5 (right-left, feet-head direction), half scan (partial Fourier) factor = 0.8×0.8 (right-left, feet-head direction), TR/TE/flip angle = 6 ms/1.97 ms/11°, acquired voxel size 1×1.4×1.6 mm^3, reconstructed voxel size 0.6×0.6×0.8 mm^3, field-of-view = 200×200×120 mm^3, scan time: 6 min 16 s). For the images shown second row, a compressed sense factor of 8 was used leading to an approximate total scan time of 4 minutes. For the third row, a compressed sense factor of 8 was used and the number of time-points were doubled to 16 (100 200 300 400 500 600 700 800 900 1000 1200 1400 1600 1800 2000 2200) leading to an approcimate total scan time of 8 minutes. In the fourth row, the compressed sense factor was reduced to 6 using the same 16 time-points leading to an approximate scan time of 11 minutes and 20 seconds.

Function

```
function [fitted_params, fitted_curve, fine_xdata] = piecewise_saturation_fit(xdata, data, lb, ub)
    % Written by Alex A. Bhogal a.bhogal@umcutrecht.nl
    % PIECEWISE_SATURATION_FIT fits a piecewise function with an initial linear period (y = 0)
    % followed by a non-linear saturating function.
    %
    % Inputs:
    %   xdata - vector of xdata points
    %   data - vector of data points corresponding to the xdata points
    %   lb - vector of lower bounds for the parameters [breakpoint, m, h]
    %   ub - vector of upper bounds for the parameters [breakpoint, m, h]
    %
    %   weights - option and can be specified to possibly improve fit
    %
    % Outputs:
    %   fitted_params - structure containing the fitted parameters
    %   fitted_curve - vector of the fitted data points
    %   fine_xdata - vector of fine-grained xdata points
  ***********************************************************************************************

    % Format data
    xdata = xdata(:);
    data = data(:);
    weights = ones(size(data));
    weights(1:3) = 4;
    weights = weights/2;
    % Initial guess for the parameters
    initial_breakpoint = mean(xdata);
    initial_m = max(data);  % Saturation level
    initial_h = mean(xdata);  % Half-saturation point

    initial_guess = [initial_breakpoint, initial_m, initial_h];
    % Objective function to minimize
    function residuals = objective(params)
        breakpoint = params(1);
        m = params(2);
        h = params(3);
        % Initialize fitted curve
        fitted_curve = zeros(size(xdata));
        % Apply the saturation model after the breakpoint
        idx_nonlinear = xdata > breakpoint;
        nonlinear_xdata = xdata(idx_nonlinear) - breakpoint;
        fitted_curve(idx_nonlinear) = m * nonlinear_xdata ./ (h + nonlinear_xdata);
        % Residuals
        residuals = (fitted_curve - data) .* sqrt(weights);
    end

    % Set options for lsqnonlin
    options = optimoptions('lsqnonlin', 'Display', 'off');
    % Perform the optimization
    fitted_params = lsqnonlin(@objective, initial_guess, lb, ub, options);
    % Extract fitted parameters
    params.breakpoint = fitted_params(1);
    params.m = fitted_params(2);
    params.h = fitted_params(3);
    % Generate the fitted curve with one data point per second
    fine_xdata = (min(xdata):1:max(xdata))';
    fine_fitted_curve = zeros(size(fine_xdata));
    idx_nonlinear = fine_xdata > params.breakpoint;
    nonlinear_xdata = fine_xdata(idx_nonlinear) - params.breakpoint;
    fine_fitted_curve(idx_nonlinear) = params.m * nonlinear_xdata ./ (params.h + nonlinear_xdata);
    % Ensure continuity at the breakpoint
    fine_fitted_curve(fine_xdata <= params.breakpoint) = 0;
    % Output the fine-fitted curve and fine-grained xdata points
    fitted_curve = fine_fitted_curve;

end
```
